# Computerized Intervention in Primary Care for Women Veterans with Sexual Assault Histories and Psychosocial Health Risks: a Randomized Clinical Trial

**DOI:** 10.1007/s11606-021-06851-0

**Published:** 2021-05-19

**Authors:** Suzannah K. Creech, Carey S. Pulverman, Christopher W. Kahler, Lindsay M. Orchowski, M. Tracie Shea, Golfo Tzilos Wernette, Caron Zlotnick

**Affiliations:** 1 VA VISN 17 Center of Excellence for Research on Returning War Veterans and the Central Texas Veterans Health Care System, Waco, TX USA; 2 Dell Medical School of the University of Texas at Austin, Austin, TX USA; 3 Department of Behavioral and Social Sciences, Brown University School of Public Health, Providence, RI USA; 4 Alpert Medical School of Brown University, Providence, RI USA; 5 Rhode Island Hospital, Providence, RI USA; 6 Providence Veterans Administration Medical Center, Providence, RI USA; 7 University of Michigan Medical School, Ann Arbor, MI USA; 8 Brown University, Providence, RI USA; 9 Women and Infants Hospital, Providence, RI USA; 10 University of Cape Town, Cape Town, South Africa

**Keywords:** sexual assault, women, hazardous drinking, intimate partner violence, posttraumatic stress disorder

## Abstract

**Importance:**

Sexual assault is a public health concern for women and is associated with subsequent psychosocial health risks of posttraumatic stress disorder (PTSD), hazardous drinking, and intimate partner violence (IPV). Sexual assault is associated with social stigma and other barriers shown to inhibit one from seeking mental health care. Digital health technologies may overcome these barriers.

**Objective:**

To test the impact of a brief computerized intervention delivered in primary care to reduce health risks and increase mental health treatment utilization among women with histories of sexual assault and current health risks.

**Design, Setting, and Participants:**

The Safe and Healthy Experiences (SHE) program was tested in a randomized controlled trial with *N* = 153 women veterans at a Veterans Health Administration (VHA) medical center, and they completed assessments at baseline, 2 months, and 4 months.

**Intervention:**

SHE is a brief motivational interviewing and psychoeducation-based computerized intervention. SHE was compared to a screen and referral-only control condition.

**Main Measures:**

Health risks were measured via validated self-report instruments. Treatment initiation and utilization were measured via self-report and chart review.

**Results:**

SHE did not impact women’s number of health risks (all *p*’s > .05). However, women randomized to SHE showed significantly greater increases in treatment use compared to women in the control group, as measured by chart review (*χ*^2^ (1, *n* = 153) = 4.38, *p* = .036, *r*_s_ = .16), and self-report (*χ*^2^ (1, *n* = 130) = 5.89, *p* = .015, *r*_s_ = .21). SHE was found to be an acceptable intervention.

**Conclusions:**

SHE was effective in improving mental health treatment initiation and utilization compared to a control group. Computer-based interventions to address sexual trauma and its consequences are acceptable, are highly scalable, and can add value to primary care with little cost or increase in provider time.

**Trial Registration:**

Clinicaltrials.gov identifier NCT02957747.

Sexual assault is highly prevalent among US women affecting an estimated 21% of women in their lifetimes ^1^. Lifetime sexual assault is associated with high rates of psychiatric disorders including posttraumatic stress disorder (PTSD) and alcohol use disorder ^2^. An unfortunate phenomenon among sexual assault survivors is the repeat experience of gender-based violence including intimate partner violence (IPV)^3^. The experience and consequences of sexual assault and the dual experience of sexual assault and IPV are accompanied by stigma and other barriers shown to delay or inhibit mental health treatment seeking when it is needed, including among women veterans ^4–8^. Women veterans experience high rates of sexual assault before, during, and after military service ^9,10^.

Numerous studies have shown the experience of sexual assault is highly stigmatized, and that societal “rape myths” that blame survivors contribute to experiences of stigma, shame, and self-blame that limit treatment seeking^8,11^. These processes have a heightened impact on marginalized populations. For example, sexual, racial, and ethnic minority women experience high rates of sexual assault, are less likely to disclose the assault^12,13^, and are less likely to seek care due to prior experiences of systemic racism and homophobia^14^ compared to other women. These barriers may be enhanced among some women veterans seeking care at VA due to the military context in which many assaults occurred, and presence of persons who may remind them of their perpetrator^15,16^, and because racial/ethnic minorities are overrepresented among women veterans compared to the general population^17^. In addition, research has shown that women veterans who identify as sexual minorities are more likely to have experienced childhood or military sexual assault compared to other women veterans ^18^.

Digital health technologies offer opportunities to reduce barriers and increase access to mental health care by delivering interventions directly to patients who need them in low- or no-stigma settings such as primary care^19^. Digital health technologies can reduce provider barriers including discomfort, lack of time, and lack of support resources ^20^ by addressing sexual assault and its consequences outside of the clinical encounter. When implemented within organized healthcare settings, health technologies offer a remedy to fragmented care and lack of care coordination^21^, potentially by screening, briefly intervening, and seamlessly referring patients to needed care. Digital mental health interventions have shown promise in overcoming barriers to care in marginalized populations, but less research has advanced to efficacy testing^19^.

Early work to develop a mobile application to address acute care needs after sexual assault has shown promise^22^; however, we are aware of no other work to address distal mental health care needs after sexual assault. This is an important gap because research shows most women do not volunteer their sexual assault history to providers ^23^, many providers do not routinely screen for sexual assault history ^24^, and most women will not present to an emergency room or mental health treatment setting in the aftermath of an assault^25,26^, often resulting in delayed or unmet mental health treatment needs. We developed the Safe and Healthy Experiences (SHE) program for women to address barriers to screening for and addressing lifetime sexual assault and related psychosocial health risks of PTSD, hazardous drinking, and IPV^27^. SHE is a modular computer-based screen and brief intervention relying on psychoeducation and the principles of motivational interviewing (MI)^28^ to reduce health risks in women with lifetime sexual trauma histories.

In a prior open trial (*N* =20), SHE was feasible for use in a Veterans Health Administration (VHA) Women’s primary care clinic, and participants reported high satisfaction with the program ^27^. The present study is a preliminary randomized controlled trial of SHE compared to a screen and referral-only control condition. Prior work in single session or single session plus booster MI-based interventions has shown these interventions are associated with reductions in hazardous drinking^29^, substance abuse and risky sex^30^, and degree of IPV^31^. Single-session telephone intervention has also been associated with better treatment engagement and PTSD symptom reduction compared to a control group^32^. Therefore, it was hypothesized that women who received SHE would evidence reductions in psychosocial health risks (primary outcome) and improved mental health care utilization (secondary outcome) compared to the control group at 2- and 4-month follow-ups. We also examined participant satisfaction with the intervention and the software.

## METHOD

### Participants

Participants were 153 women veterans seeking primary care at a VHA medical center. Inclusion criteria were self-identified female gender, age between 18 and 65, a history of sexual assault (defined as at least one incident of unwanted lifetime sexual contact), and at least one current psychosocial health risk (PTSD, hazardous drinking, and/or IPV). Exclusion criteria were as follows: inability to understand study procedures in English, active suicidal or homicidal crisis warranting imminent clinical intervention. The study was approved by the Institutional Review Board and occurred from May, 2017, through April, 2019. De-identified summary data may be available upon request pending institutional approvals.

### Procedure

#### Recruitment

**Figure 1:**
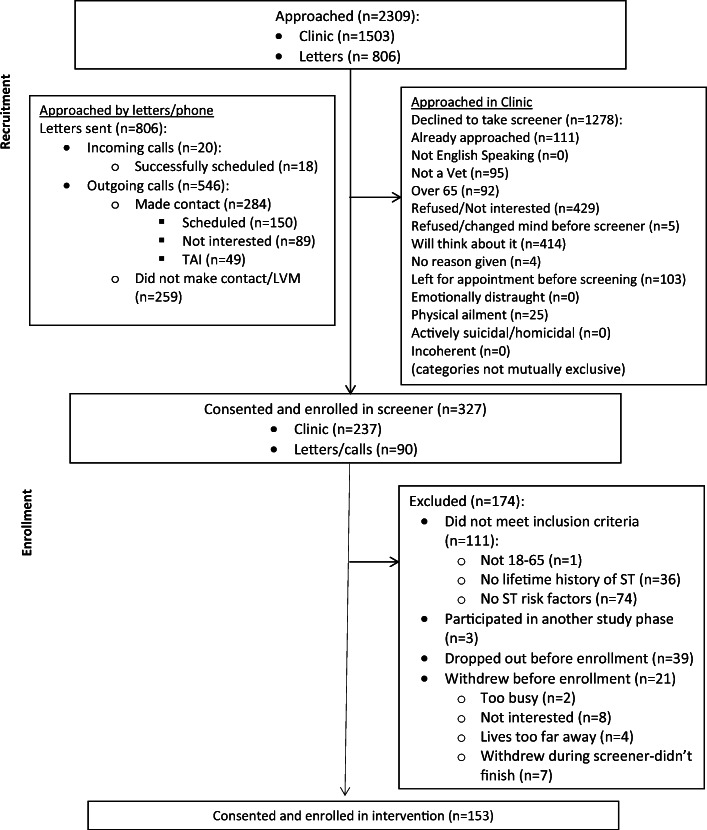

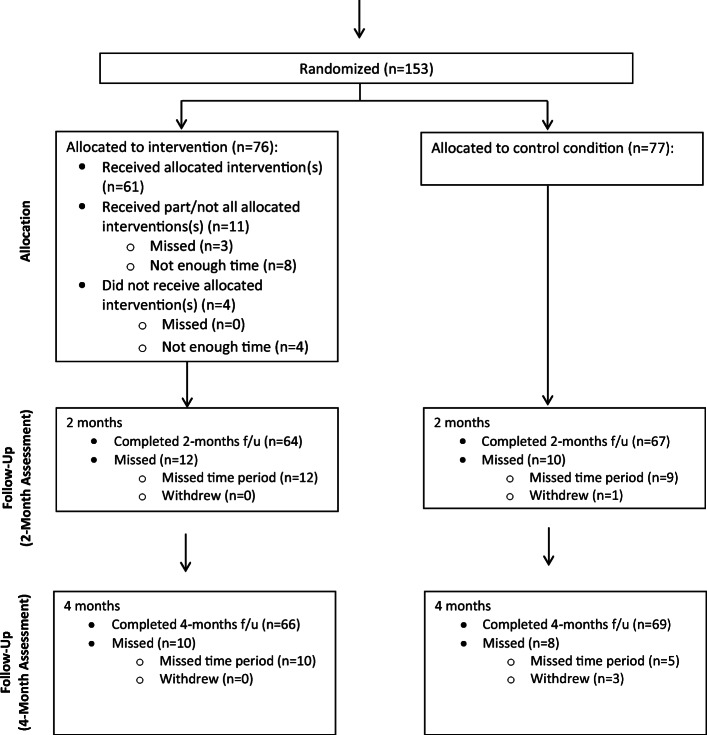
CONSORT table for the randomized controlled trial of the SHE intervention.

The study was advertised via fliers, in-person recruitment in women’s primary care clinics, and letters to all women who had primary care appointments scheduled in the next month (see Fig. 1).

#### Screening Assessment

Interested participants were presented with study details, provided written informed consent, and completed self-report screening measures on an iPad™ in a private research office. At the conclusion, all participants were provided with a list of mental health and IPV resources within and outside the VA. Eligible women were invited to participate in the randomized controlled trial. Participants were compensated monetarily by gift cards for time spent completing screening ($10) and assessments ($30, $40, and $50 for the baseline, 2-month and 4-month follow-ups). All participants screened were also entered into a raffle for $100 that was conducted every 100 participants.

#### Baseline Assessment

Women (*N* = 153) completed a self-report baseline assessment and were randomized to the intervention or control condition using a standard randomization procedure within the computerized software. After completion of the baseline assessment, the (computer) narrator “flipped a coin” and women (*N*=153) were randomized into the control or SHE intervention. The randomization sequence was known only to the computer program and optimized for balanced assignment over time between the two conditions. This procedure resulted in *n* = 76 assigned to the intervention and *n* = 77 assigned to the control condition.

#### Control Procedure

Those randomized to the control condition completed assessments only. After baseline, they were offered a list of mental health and IPV referrals and resources. They were assisted with referrals directly any time throughout the study if requested.

#### Intervention Procedure

Those randomized to the intervention were presented with the module(s) for each relevant health risk (i.e., PTSD, hazardous drinking, and/or IPV). Modules took about 20 min each and included an audio-visual presentation on the iPad™ and corresponding psychoeducational resource handouts. At the conclusion, they were offered a list of mental health and IPV referrals and resources and assisted with referrals directly any time throughout the study if requested.

#### Intervention Content

SHE was developed using the Computerized Intervention Authoring System (CIAS) ^33^. Each SHE intervention module presented personalized feedback from the baseline assessment. Next the modules included psychoeducational information on the health risk endorsed and brief videos of women speaking about that health risk and their own experiences and utilization of resources and/or skills to reduce their risk. Materials were tailored to diverse women who had served in the military. Questions based on motivational interviewing and the stages of change model ^28^ were presented. Women reporting high readiness to change (e.g., Yes, I am ready to take steps toward my health) engaged in a goal-setting process using resources from SHE to plan steps toward their goals. Women reporting ambivalence about change (e.g., No, I don’t think that I’m ready to take those steps) received targeted feedback, a list of sample pros and cons about change, and information and videos about steps toward change (e.g., building support, self-talk, seeking treatment, evidence based treatment, using resources, safety planning). Modules ended with a motivational video and feedback survey.

#### Two-Month and Four-Month Follow-Up Assessments

Women completed in-person follow-up assessments on the iPad^TM^ at 2 and 4 months from the date of their baseline assessment. A subset of the sample (< 5%) completed follow-ups by phone due to moving out of the area or unavailability. All follow-ups were scheduled at the end of the baseline assessment, and women received reminder calls and letters shortly before each follow-up and for any missed follow-ups.

### Materials

#### Screening Measures

Women completed brief screening measures on history of sexual assault, PTSD, hazardous drinking, and IPV. Experiences of sexual assault were assessed with the Childhood Sexual Victimization Questionnaire (CSVQ) ^34^, and the Sexual Experiences Survey–Short Form Victimization (SES-SFV) ^35^, and a military sexual assault screener developed by the VHA ^36,37^. PTSD was assessed with the PTSD Checklist for Diagnostic and Statistical Manual of Mental Disorders–Fifth Edition (PCL-5)^38,39^. Hazardous drinking was assessed with the Graduated Frequency Measure (GFM) ^40^, a measure of alcohol use in the past month and a positive screen was indicted by having four or more drinks on any single day in the past month ^41,42^. IPV in the past year was assessed with the Woman Abuse Screening Tool (WAST) ^43^. A positive screen for IPV was indexed by a score of four or greater on the WAST.

#### Baseline, Two-Month, and Four-Month Assessment Measures

Participants completed assessments on PTSD, hazardous drinking, IPV, and treatment utilization at the baseline, 2-month, and 4-month assessments. Differences between screening measures and assessment measures are because the screening needed to be completed in approximately 10 min or less, which required shorter measures for drinking and IPV.

#### PTSD

Respondents rated past month PTSD symptom severity on the 20-item PCL-5^38,44^. Higher scores reflect greater symptoms. A score of 33 is the clinical cutoff for probable PTSD and was used as an indicator of PTSD as a health risk^39^. The PCL-5 has excellent psychometric properties^39^. Internal consistency across all administrations in this sample ranged from *α* = .95 to .96.

#### Hazardous Drinking

The 10-item Alcohol Use Disorder Identification Test (AUDIT) ^45^ was used to assess hazardous drinking as a health risk. At baseline, this was assessed for the past year; at follow-ups, this was assessed for the past 2 months. Higher scores reflect greater alcohol use and a score of eight is the cutoff for hazardous drinking^46^. The AUDIT possesses high test-retest reliability and good internal consistency ^47,48^. Internal consistency across all administrations in the current sample ranged from *α* = .85 to .88.

#### IPV

The Composite Abuse Scale (CAS) is a 30-item measure of IPV in the past 12 months ^49^. Higher scores indicate more abuse and a score of 3 or more was used as an indicator that IPV was a health risk. The CAS has evidenced good psychometric properties ^49^. Internal consistency across all administrations in the current sample ranged from *α* = .91 to .95. At follow-ups, participants reported on the past 2 months.

#### Treatment Use

The Treatment Services Review is a 15-item measure of health care treatment use ^50^ and was used to assess self-report of mental health treatment in the past 2 months (individual and group therapy, 12-step group sessions, residential substance abuse treatment, psychological testing, inpatient psychiatric care; one day of residential or inpatient psychiatric care was treated as one unit of care). This measure is reported as the total number of mental health care appointments. Study staff also reviewed each participant’s medical record and tallied mental health care appointments in the prior 2 months at baseline and follow-ups.

#### Intervention Feedback

The CIAS Software Scale (SCSS^33^) was used to assess participant satisfaction with the software in terms of likeability, ease of use, interest, and respectfulness. Items are evaluated on a 5-point Likert scale (1 = *not at all*, and 5 = *very much*). Internal consistency for the CIAS in the current sample was good (*α* = .77). Intervention feedback was assessed with the 8-item Client Satisfaction Questionnaire on participant satisfaction with the intervention (CSQ^51^). Internal consistency for the CSQ in the current sample was also excellent (*α* = .90).

#### Data Analysis

All analyses use the full intent-to-treat sample. To test our primary hypothesis, we first tested between group effects on the number of health risks present at both 2 months and 4 months using Mantel-Haenszel chi-square tests. We used Spearman rank-order correlations to depict the strength of the association between treatment groups and number of risks. To examine the effect of treatment group over time while controlling for baseline risks, we then ran regression models predicting the number of risks at each follow-up controlling for the number of health risks at baseline. Treatment group was dummy coded with control group as the reference group, and a Poisson distribution with a logit link function was specified for this count outcome.

For treatment utilization during follow-up, the outcome data both from chart review and the TSR were highly positively skewed with some zero inflation. To represent these data in a clinically meaningful way, we categorized each participant’s treatment utilization across the 4 months of follow-up into one of the following four levels: no treatment attended; attended treatment up to once a month on average; attended treatment more than once a month on average but not more than weekly; and attended treatment more than weekly. We then used Mantel-Haenszel chi-square tests to determine whether conditions differed significantly at follow-up on these categorical service utilization outcomes with the hypothesis that those receiving SHE would be more likely to be classified as having higher utilization compared to those in control. To depict the strength of the association between treatment group and level of treatment utilization, we calculated Spearman rank-order correlations. We then used logistic regressions to predict utilization level adjusting for the respective value of the outcome at baseline. Ordered logistic regression was used to test whether SHE compared to control was associated with greater odds of being in a higher level of treatment utilization. We also examined satisfaction with the intervention and software as well as intervention effects on raw scores for PCL-5, AUDIT, and CAS.

#### Sample Size Determination

Sample size was set to detect a medium effect size across primary outcomes with power of .85 using an alpha of .05. To achieve this power to detect a medium effect size of *w* = .30, a final sample size of 122 was required for a categorical outcome with three levels such as the number of risks. To allow for 20% loss to follow-up, we set our desired sample size at 150 participants.

## RESULTS

### Demographics

Participant ages ranged from 24 to 65 with a mean of 43.55 (*SD* = 10.10). The sample was diverse, and the majority of participants identified as non-Hispanic and African American/Black or White (see Table 1).

**Table 1:** Demographics of Study Sample (*N* = 153)

	Full sample		Control group		Intervention group	
	*M* (SD)	Range	*M* (SD)	Range	*M* (SD)	Range
Age	43.55 (10.10)	24–65	43.63 (10.28)	24–65	43.46 (9.97)	26–62
Sexual orientation						
Heterosexual	132	86.3	64	83.1	68	89.5
Bisexual	7	4.6	2	2.6	5	6.6
Lesbian	10	6.5	7	9.1	3	3.0
Other	4	2.6	4	5.2	0	0.0
Ethnicity						
Not Hispanic/Latina	130	85	65	84.4	65	85.5
Hispanic/Latina	23	15	12	15.6	11	14.5
Race						
African American/Black	73	47.7	37	48.1	36	47.4
White	54	35.3	26	33.8	28	36.8
Bi-racial/multi-racial	10	6.5	8	10.4	2	2.6
Asian	2	1.3	0	0.0	2	2.6
Native American or Alaska Native	2	1.3	1	1.3	1	1.3
Native Hawaiian or Other Pacific Islander	1	.7	0	0.0	1	1.3
Other	9	5.9	4	4.2	5	6.6
Decline to answer	2	1.3	1	1.3	1	1.3
Education						
High school/GED	12	7.8	10	13.0	2	2.6
Technical/trade school	12	7.8	2	2.6	10	13.2
Some college	62	40.5	31	40.3	31	40.8
College graduate	50	32.7	24	31.2	26	34.2
Postgraduate	17	11.1	10	13.0	7	9.2
Relationship status						
Married	53	34.6	27	35.1	26	34.2
Separated	10	6.5	6	7.8	4	5.3
Divorced	54	35.3	24	31.2	30	39.5
Single, no relationship	20	13.1	8	10.4	12	15.8
Single, in a relationship	16	10.5	12	13.0	4	5.3
Sexual trauma history						
Unwanted sexual contact childhood	89	58.2	46	59.7	43	56.6
Any adulthood sexual assault	118	77.1	75	97.4	76	100.0
Adulthood sexual assault	11	7.2	8	10.4	3	3.9
Sexual assault during military service	107	69.9	54	70.1	53	69.7

### Number of Health Risks (Primary Outcome)

Number of health risks at baseline, 2 months, and 4 months are shown in Table 2. At baseline, results of Mantel-Haenszel chi-square tests indicated that the group difference in number of risks was just over the .05 significance level, *χ*^2^ (1, *n* = 152) = 2.82, *p* = .09; effect size *r*_s_ = .13, with those in the SHE group reporting a higher number of risks. At 2 months, the linear association between group and number of risks was significant and participants assigned to SHE were more likely to have a higher number of risks, *χ*^2^ (1, *n* = 117) = 3.94, *p* = .047; *r*_s_ = .20. This association was nonsignificant at 4 months, *χ*^2^ (1, *n* = 133) = 0.97, *p* = .32; *r*_s_ = .10. Results of Poisson regression analyses adjusting for number of risks at baseline indicated that the effect of intervention condition was nonsignificant at both 2 months (incidence rate ratio [IRR] = 1.18, 95% CI [0.84, 1.65], *p* = .34) and 4 months (IRR = 1.07, 95% CI [0.77, 1.51], *p* = .66).

**Table 2:** Baseline, 2-Month, and 4-Month Health Risks by Treatment Group

Time	PTSD^a^		Hazardous drinking^b^		IPV^c^		Total
	*M* (SD)	Health risk, *n*/*N* (%)	*M* (SD)	Health risk, *n*/*N* (%)	*M* (SD)	Health risk, *n*/*N* (%)	Number of risks *M* (SD)
Control group							
Baseline	49.71 (17.44)	44/77 (57.14)	5.83 (5.79)	22/76 (28.94)	12.31 (17.34)	42/77 (54.54)	1.39 (.88)
2 months	35.81 (20.82)	32/57 (56.14)	5.27 (5.60)	12/59 (20.34)	4.32 (8.16)	12/59 (20.34)	1.05 (.93)
4 months	37.52 (20.64)	40/69 (57.97)	4.42 (5.01)	14/69 (20.29)	3.98 (8.96)	16/68 (23.53)	1.00 (.81)
Intervention group							
Baseline	51.91 (16.95)	59/76 (77.63)	5.55 (5.15)	22/76 (28.94)	14.96 (19.92)	42/76 (55.26)	1.62 (.75)
2 Months	43.58 (18.54)	42/60 (70.0)	4.65 (5.07)	15/62 (24.19)	8.43 (12.79)	28/60 (46.67)	1.38 (.85)
4 Months	41.39 (18.07)	44/65 (67.69)	4.52 (5.54)	10/66 (15.15)	5.00 (9.75)	21/66 (31.81)	1.14 (.81)

^a^PTSD symptoms were assessed with the PTSD Checklist for DSM-5 and the PTSD health risk was defined as a score greater than or equal to 33

^b^Drinking behavior was assessed with the Alcohol Use Disorder Identification Test (AUDIT) and the hazardous drinking health risk was defined as a score of greater than or equal to eight

^c^IPV was assessed with the Composite Abuse Scale (CAS) and the IPV health risk was defined as a score greater than or equal to three

### Treatment Use (Secondary Outcome)

Table 3 shows the levels of frequency of treatment receipt by chart review and self-report at baseline and across follow-up. Results of Mantel-Haenszel chi-square tests indicated that at follow-up, the linear association between group and level of treatment engagement was significant, where participants in SHE compared to those in control were more likely to have higher levels of treatment as assessed by both chart review (*χ*^2^ (1, *n* = 153) = 4.38, *p* = .036, *r*_s_ = .16), and self-report (*χ*^2^ (1, *n* = 130) = 5.89, *p* = .015, *r*_s_ = .21). Differences by group were nonsignificant in the 3 months prior to baseline (*χ*^2^ (1, *n* = 153) = 0.29, *p* = .59, *r*_s_ = .05, and *χ*^2^ (1, *n* = 152) = 1.56, *p* = .21, *r*_s_ = .10) for chart review and self-report, respectively. Results of ordered logistic regression analyses, which adjusted for baseline, indicated that receiving the SHE intervention compared to control was associated with greater odds of being classified in a higher level of treatment receipt, odds ratio [OR] = 2.17, 95% CI [1.11, 4.24], *p* = .02. For level of treatment receipt by self-report, there was a trend toward higher levels of treatment for the intervention group [OR] = 1.67, 95% CI [0.92, 3.04], *p* = .09. Levels of treatment receipt at follow-up are shown in Table 4. The most notable change in the SHE group was among those who did not receive any treatment at baseline, most of whom received at least some treatment during follow-up. This change was much less pronounced in the control group.

**Table 3:** Treatment Utilization from Baseline to 4-Month Follow-up

Treatment frequency	Chart review baseline, *n*/*N* (%)	Chart review follow-up, *n*/*N* (%)	Treatment Services Review baseline, *n*/*N* (%)	Treatment Services Review follow-up, *n*/*N* (%)
Control				
Never	29/77 (37.66)	22/77 (28.57)	22/76 (28.95)	19/67 (28.36)
Up to once monthly	28/77 (36.36)	26/77 (33.77)	25/76 (32.89)	19/67 (28.36)
>Monthly up to weekly	16/77 (20.78)	23/77 (29.87)	22/76 (28.95)	23/67 (34.33)
More than weekly	4/77 (5.19)	6/77 (7.79)	7/76 (9.21)	6/67 (8.96)
Intervention				
Never	25/76 (32.89)	10/76 (13.16)	17/76 (22.37)	10/63 (15.87)
Up to once monthly	29/76 (38.16)	31/76 (40.79)	23/76 (30.26)	15/63 (23.81)
>Monthly up to weekly	18/76 (23.68)	23/76 (30.26)	26/76 (34.21)	24/63 (38.10)
More than weekly	4/76 (5.26)	12/76 (15.79)	10/76 (13.16)	14/63 (22.22)

Treatment use was assessed with a chart review of the VHA medical record and via self-report with the Treatment Services Review measure. To represent these data in a clinically meaningful way, we categorized each participant’s treatment utilization in each time period into one of the following four levels: no treatment attended; attended treatment up to once a month on average; attended treatment more than once a month on average and but not more than weekly; and attended more than weekly. The baseline time period referred to the two months prior to the baseline appointment. The follow-up time period referred to the four months of follow-up during the study

**Table 4:** Relationship Between Treatment Use at Baseline and Treatment Use at Follow-up

Treatment frequency at BL	Follow-up never, *n*/*N* (%)	Follow-up up to once monthly, *n*/*N* (%)	Follow-up >monthly up to weekly, *n*/*N* (%)	Follow-up more than weekly, *n*/*N* (%)
Chart review				
Control				
BL never	22/29 (75.86)	7/29 (24.14)	0/29 (0.0)	0/29 (0.0)
BL up to once monthly	0/28 (0.0)	17/28 (60.71)	11/28 (39.29)	0/28 (0.0)
BL >monthly up to weekly	0/16 (0.0)	2/16 (12.5)	12/16 (75.0)	2/16 (12.5)
BL more than weekly	0/4 (0.0)	0/4 (0.0)	0/4 (0.0)	4/4 (100.0)
Intervention				
BL never	10/25 (40.0)	12/25 (48.0)	2/25 (8.0)	1/25 (4.0)
BL up to once monthly	0/29 (0.0)	17/29 (58.62)	10/29 (34.48)	2/29 (6.90)
BL >monthly up to weekly	0/18 (0.0)	2/18 (11.11)	11/18 (61.11)	5/18 (27.78)
BL more than weekly	0/4 (0.0)	0/4 (0.0)	0/4 (0.0)	4/4 (100.0)
Treatment Services Review				
Control				
BL never	15/18 (83.33)	1/18 (5.56)	2/18 (11.11)	0/18 (0.0)
BL up to once monthly	2/21 (9.52)	10/21 (47.62)	9/21 (42.86)	0/21 (0.0)
BL >monthly up to weekly	2/21 (9.52)	5/21 (23.81)	11/21 (52.38)	3/21 (14.29)
BL more than weekly	0/6 (0.0)	2/6 (33.33)	1/6 (16.67)	3/6 (50.0)
Intervention				
BL never	5/13 (38.46)	5/13 (38.46)	3/13 (23.08)	0/13(0.0)
BL up to once monthly	4/19 (21.05)	6/19 (31.58)	8/19 (42.11)	1/19 (5.26)
BL >monthly up to weekly	1/22 (4.54)	4/22 (18.18)	10/22 (45.45)	7/22 (31.82)
BL more than weekly	0/9 (0.0)	0/9 (0.0)	3/9 (33.33)	6/9 (66.67)

Table presents participants’ treatment use level (i.e., no treatment attended; attended treatment up to once a month on average; attended treatment more than once a month on average and but not more than weekly; and attended more than weekly) at baseline and at follow-up to illustrate the number of women whose treatment use changed across time. Treatment use included mental health and substance use treatment appointments only. Significantly more women in the intervention group advanced in their treatment use level than women in the control group

### Treatment Satisfaction and Change in Raw Scores

Software satisfaction ratings (Table 5) as measured by the SCSS were high *n* = 95, *M* = 4.42, *SD* = 0.56, range = 2.86–5. Treatment satisfaction as measured by the CSQ was moderately high and very similar across each of the modules: PTSD (*n* = 48, *M* = 3.28, range = 2.13–4, *SD* = 0.51); alcohol use (*n* = 23, *M* = 3.18, range = 2.13–4, *SD* = 0.52); IPV (*n* = 31, *M* = 3.35, range = 2.38–4, *SD* = 0.47).

**Table 5:** Mean Ratings on Satisfaction with the SHE Intervention

	*M* (SD)
Satisfaction with CIAS Software Scale (SCSS)^a^	
How much did you like it?	4.39 (.84)
How interesting was it?	4.33 (.99)
Was it easy to use?	4.84 (.45)
How understandable was it?	4.82 (.46)
How respectful of you was it?	4.88 (.35)
How annoyed by it were you?*	3.52 (1.40)
How interested are you in using the software again in the future?	4.14 (1.06)
Ratings on the Client Satisfaction Questionnaire (CSQ)^b^	
How would you rate the quality of the service you received?	3.63 (.49)
Did you get the kind of services you wanted?	3.41 (.54)
To what extent has our program met your needs?	2.95 (.79)
If a friend were in need of similar help, would you recommend our program?	3.41 (.54)
How satisfied are you with the amount of help you received?	3.21 (.74)
Have the services you received helped you to deal more effectively with your problems?	3.07 (.73)
In an overall, general sense, how satisfied are you with the services you received?	3.26 (.69)
If you were to seek help again, would you come back to our program?	3.31 (.60)

^a^The Satisfaction with CIAS Software Scale (SCSS) is rated on a 5-point Likert Scale from 1 = *not at all* to 5 = *very much*

*This item was reverse scored such that higher scores indicate *lower* annoyance with the software program

^b^The Client Satisfaction Questionnaire (CSQ) is rated on a 4-point Likert Scale, with higher numbers indicating greater satisfaction. Participants completed these questionnaires after *each* module; thus, women with multiple risk factors completed these measures in reference to each module respectively. The satisfaction questionnaires were completed a total of 95 times by 72 participants

Means on the PCL-5, AUDIT, or CAS by condition were examined. Results of *t* tests did not indicate any advantage for the SHE group relative to control at 2 or 4 months on any of these three continuous outcomes with all *p*-values >.25 except for the CAS at 2 months. For the CAS at 2 months, those in the SHE cohort reported significantly more interpersonal violence than those in the control, *t*(115) = 2.06, *p* = .04.

## DISCUSSION

The primary goal of this study was to test the impact of a computerized screener and brief intervention delivered in primary care on number of health risks and mental health treatment utilization among women with histories of sexual assault and positive screens for PTSD, hazardous drinking, or IPV. There was no effect of the SHE intervention on number of psychosocial health risks reported during follow-up. However, we found support for our hypothesis that women in the SHE group would evidence improved mental health care utilization compared to the control group. At both 2- and 4-month follow-ups, women in the SHE group had higher rates of treatment initiation and utilization. Findings have important clinical significance for the serious public health concern of sexual assault against women, which is associated with psychiatric disorders^2^, low utilization of mental health treatment^4–7^, and increased risk for re-victimization^3^. The SHE program provides a promising tool for engaging women in mental health treatment that overcomes several provider and patient barriers and has the potential to have a high reach.

Prior research has consistently shown that sexual assault is a highly stigmatized experience and this stigma is a significant contributor to barriers to mental health treatment seeking^8^. Considering the socio-political context in which sexual assault often occurs, it is not surprising most women do not volunteer their sexual assault history to providers ^23^, many providers do not routinely screen for sexual assault history ^24^, and most women will not present to an emergency room or mental health treatment setting in the aftermath of an assault^25,26^. Sexual, racial, and ethnic minority women experience high rates of sexual assault and are less likely to disclose the assault^12,13^ and to seek care due to prior experiences of systemic racism and homophobia^13,14,52^. Among women veterans who experienced sexual assault in the military, the intersection between gender, race, and sexual identities^53^ may contribute to enhanced barriers to care after sexual assault at VA due to institutional reminders of the military context in which many assaults occurred^15,16^. Although further efficacy testing is needed, findings from this study lend support to the growing evidence that digital mental health technologies can overcome barriers to care in marginalized populations^19^.

Other goals of the study were to examine the efficacy of SHE in reducing women’s overall number of psychosocial health risks, as well as symptoms of PTSD, hazardous drinking, and recent experiences of IPV compared to the control group. Although other single-session interventions have shown positive effects on drinking, IPV, and PTSD ^29–32^, there were no differences in any of these measures between the SHE and control groups, potentially because the study was powered to detect only a medium effect size when most behavioral interventions yield small- to medium-range effect sizes^54^. Moreover, the study was underpowered to test mediation models, and any impact on clinical outcomes would likely be through treatment utilization. The follow-up period (4 months) was also short in comparison to the time it may take to observe changes in these health risks considering the high levels of symptom severity and comorbidity in the sample. A final confounding factor is the lack of specificity in our measurement of the type of mental health treatment received. For example, women may have sought treatment for a different condition that we did not measure. Patient education on evidence-based treatment for mental health problems has been shown to result in better outcomes^55^, and our lack of measurement of both condition treated and receipt of adequate dose of evidence-based care could have obscured findings. These possibilities should be examined in a future study, with an extended follow-up period and a larger sample size powered to test mediation.

## LIMITATIONS

Limitations of the study include a low sample size to detect small effect sizes or to test mediation models, a low N in the alcohol group, and a relatively short follow-up period. We also tested SHE with women veterans seeking primary care at the VA, which is an optimal environment to test a computerized intervention to reduce health risks and improve referral to mental health care among women with sexual assault histories but may limit generalizability. Although we pre-specified our primary and secondary outcomes, the total number of analyses reported for number of health risks and treatment use (including both chart review and self-report) was large given that we examined health risks at both 2 and 4 months and presented both unadjusted and adjusted treatment effects for all outcomes. Thus, there was some inflation of type I error risk due to this multiple testing. Limitations notwithstanding, strengths of this study include a rigorous, randomized design, and enrollment of a diverse sample of women.

## CONCLUSIONS

To our knowledge, this is the first clinical trial of a modular, computer-based screen, and brief intervention delivered in a primary care setting to address prevalent consequences of sexual assault for women. Although it did not change health risks, SHE was acceptable among women veterans within a primary care setting and improved mental health treatment initiation and utilization compared to a control group. Further study of SHE in a fully powered confirmatory efficacy trial should be conducted. Computer-based interventions to address sexual assault and its consequences appear acceptable, are highly scalable, and can add value to primary care with little increase in provider time. Moreover, computer-based interventions may be uniquely well suited to addressing barriers to care in marginalized populations.
